# Effectiveness of regorafenib in second-line therapy for advanced hepatocellular carcinoma: A systematic review and meta-analysis

**DOI:** 10.1097/MD.0000000000041356

**Published:** 2025-01-24

**Authors:** Yunzhi Shen, Yu Bai

**Affiliations:** a Department of Hepatobiliary Surgery, The Third Central Hospital of Tianjin, Tianjin, China; b Tianjin Key Laboratory of Extracorporeal Life Support for Critical Diseases, Tianjin, China; c Artificial Cell Engineering Technology Research Center, Tianjin, China; d Tianjin Institute of Hepatobiliary Diseases, Tianjin, China.

**Keywords:** cabozantinib, Hepatocellular carcinoma, regorafenib, second-line therapy, sorafenib

## Abstract

**Background:**

In patients with advanced hepatocellular carcinoma (HCC) following sorafenib failure, regorafenib has been used as an initial second-line drug. It is unclear the real efficacy and safety of sorafenib-regorafenib sequential therapy compared to placebo or other treatment (cabozantinib or nivolumab or placebo) in advanced HCC.

**Methods:**

Four electronic databases (PubMed, Embase, Web of Science, and Ovid) were systematically searched for eligible articles from their inception to July, 2024. Included articles were selected based on strict eligibility criteria. Review Manager 5.4 software were performed for statistical analysis.

**Results:**

Ten studies with 2349 HCC patients of whom 1370 received regorafenib treatment, and 979 underwent cabozantinb, nivolumab or placebo were selected for meta-analysis. The results of our systematic review and meta-analysis found regorafenib could significantly prolong overall survival (standardized mean difference [SMD] = 0.16, 95% CI = 0.02 to 0.29, *P* = .02) than other treatment (cabozantinib or nivolumab or placebo) in second-line treatment of HCC following sorafenib failure. No significant difference in progression-free survival (SMD = −0.03; 95% CI = −0.13 to 0.06; *P* = .53), overall response rate (risk ratio [RR] = 0.59; 95% CI = 0.24 to 1.47; *P* = .26), disease control rate (RR = 1.23; 95% CI = 0.7 to 2.16; *P* = .48) between 2 groups. Subgroup analysis demonstrated that nivolumab has better overall response rate results than regorafenib (RR = 0.38; 95% CI = 0.24 to 0.61; *P* < .0001).

**Conclusions:**

Compared with other treatment (cabozantinib or nivolumab or placebo), regorafenib seemed to be more effective for patients with HCC who have not responded to initial sorafenib treatment.

## 1. Introduction

Hepatocellular carcinoma (HCC) is the third leading cause of cancer-related death and the 6 highest incident cancer in the world.^[1]^ It is a malignant tumor with poor prognosis due to the few specific or evident symptoms in early stage, and most HCC patients are diagnosed with advanced cancer. At the initial diagnosis, <30% of patients could undergo curative treatment, such as hepatectomy, radiofrequency ablation (RFA), or transarterial chemoembolization (TACE).^[2,3]^ Despite progress in early detection, most patients with HCC are still diagnosed with advanced cancer.^[4]^

Sorafenib is an oral multikinase inhibitor that target various protein receptors, including vascular endothelial growth factor receptor (VEGFR) and platelet-derived growth factor receptor (PDGFR), to impair vascular angiogenesis. It also blocks various cells signaling pathways, such as Raf-1 proto-oncogene, B-Raf proto-oncogene, and kinase activity in the MAPK/ERK signaling pathways to inhibit tumor progression and angiogenesis and promotes tumor cell apoptosis.^[5]^ Sorafenib was the first oral small-molecule tyrosine kinase inhibitor to be approved as a first-line treatment for advanced HCC. In 2007, it was established as the standard treatment for patients with advanced HCC, following the positive results of 2 randomized phase III trials.^[6,7]^ However, the majority of patients with advanced HCC do not experience long-term disease control with initial systemic therapy, and about 40% to 56% patients receive second-line therapy due to treatment failure.^[8]^

Several drugs have been tested as second-line treatments for sorafenib-resistant patients,^[9–11]^ such as Pegargiminase, Bevacizumab, and Tivantinib, but no positive results were obtained before until the RESORCE trial.^[12]^ The results of the RESORCE trial demonstrated that patients in the regorafenib group had a 37% lower mortality risk compared to those in the control group. The median overall survival (OS) of patients in the regorafenib group was 10.6 months, while in the placebo group it was 7.8 months, with a hazard ratio (HR) of 0.63.^[12]^ Regorafenib is a multikinase inhibitor that targets various kinases involved in tumor growth and progression, including those involved in angiogenesis, tumorigenesis, and the tumor microenvironment.^[13]^ It has been established as a standard second-line treatment and is often used in a sequential treatment plan with sorafenib for patients with advanced HCC.

In this study, we systematically reviewed all articles that compared the sorafenib-regorafenib sequential therapy to placebo or other therapy (cabozantinib or nivolumab) in advanced HCC.

## 2. Materials and methods

The manuscript was written in accordance with the Preferred Reporting Items for Systematic reviews and Meta-Analyses (PRISMA) guidelines for protocols.^[14]^ In this study, ethical approval is unnecessary as it is a meta-analysis of existing literatures and does not address data from individual patients.

### 2.1. Search strategy

A systematic search was performed in electronic databases, including PubMed, Embase, Web of Science, and Ovid. The search terms were as follows: “regorafenib,” “hepatocellular cancer,” “liver cancer,” “hepatocellular carcinoma” and their synonyms or similar words (from their inception to July, 2024). Searches were restricted to English-language literature and was first screened by 2 independent reviewers (YS and YB) who reviewed the titles of the papers and available abstracts. In addition, a manual search was conducted on the reference list of all included literatures and related reviews to found other studies that may meet the criteria.

### 2.2. Inclusion and exclusion criteria

For inclusion, literatures were screened based on the following criteria: study design was randomized controlled trials or retrospective study; patients who are 18 years of age or older and have advanced HCC; second-line systemic treatment for HCC; the treatment group was regorafenib.

Studies were excluded if they met any of the following criteria: combined with other diseases; did not have a control group; abstracts, letters, or case–control studies.

### 2.3. Assessment of methodological quality of included articles

Evaluating the literatures quality using the Cochrane Collaboration risk of bias tools.^[15]^ The evaluation results were measured in the following aspects: generation of random sequences, allocation concealment, blinding by researchers and patients, blinding by outcome assessors, incomplete outcome measures, and selective reporting.

### 2.4. Data extraction

Data were extracted from each article: publish year, study design, therapeutic regimens, sample size, age and sex ratio, number of treatment lines, overall survival (OS), progression-free survival (PFS), overall response rate (ORR) and disease control rate (DCR).

### 2.5. Statistical analysis

The statistical analysis was conducted by Review Manager 5.4. The outcome measures of the meta-analysis were presented using risk ratio (RR), standard mean difference (SMD), and 95% confidence interval (CI). Heterogeneity testing was performed on each study using the chi-square test. If there was no statistical heterogeneity between studies (*P* > .05, *I*^2^ ≤ 50%), a fixed effects model was performed for the meta-analysis. However, if there was heterogeneity between studies (*P* < .05, *I*^2^ > 50%), a random-effects model was used. In addition, subgroup analysis and sensitivity analysis were conducted to identify the source of heterogeneity.

## 3. Results

### 3.1. Search results and bias

**Figure 1.**
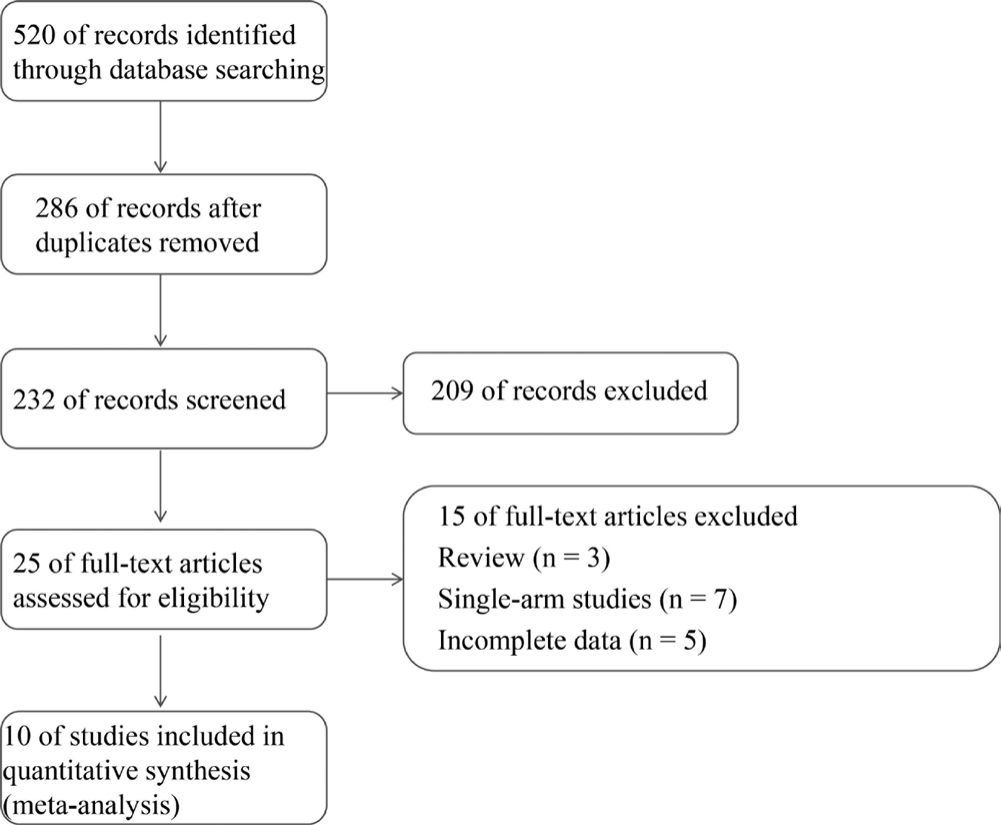
Flow chart showing results of the literature search and study inclusion. The process followed the PRISMA guidelines. PRISMA = Preferred Reporting Items for Systematic reviews and Meta-Analyses.

The literature search process was shown in Figure 1. The initial search yielded 520 potentially related researches. After removing duplicates (n = 286) and filtering abstracts (n = 209), the eligibility of 25 full-text studies was evaluated. Out of these, 15 studies were excluded for various reasons: 3 were reviews, 7 were single-arm studies, and 3 had incomplete data. A total of 10 publications^[2,12,16–23]^ ultimately met the criteria for the final meta-analysis. Among them, “Bruix et al” and “Finn et al” reported different data from the same study. One study was an RCT^[12]^ and 9 retrospective studies. No other citations were found in references.

**Figure 2.**
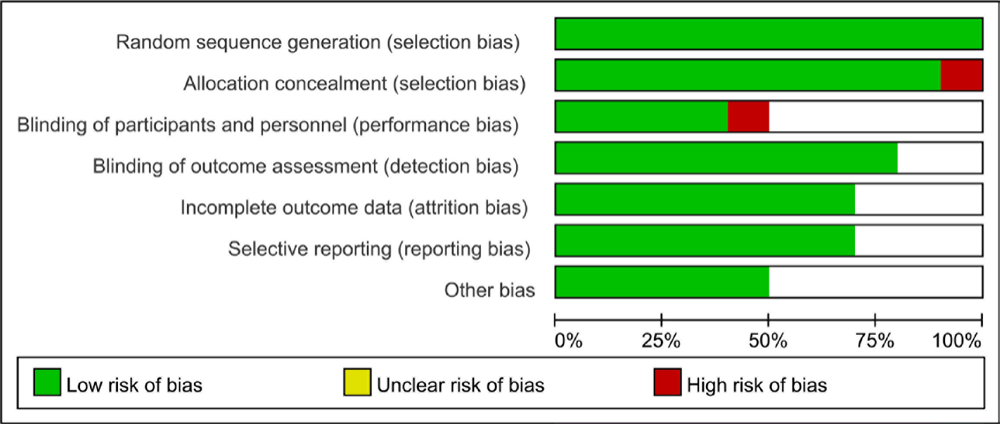
Risk of bias assessment in randomized trials and single-arm studies. Green indicates low risk of bias, yellow indicates medium risk of bias, and red indicates high risk of bias.

Figure 2 summarizes the detailed information on the bias assessment risk of the included studies. According to appropriate random sequences, all studies were evaluated as low risk.

### 3.2. Study characteristics

A total of 10 studies including 2349 patients were identified available in the meta-analysis. All included patients were refractory patients who were resistant or intolerant to sorafenib. Of all the 2349 patients, 1370 received regorafenib treatment, and 979 underwent cabozantinb, nivolumab, or placebo. The baseline clinic-pathological features of the included studies were presented in Table 1.

**Table 1:** Main characteristics of the studies included in the meta-analysis.

	Included studies	studies design	Treatment group	Control group	Sample size	Age, years	Sex (male/female)	Line
1	Adhoute 2022	Retrospective multicenter study	Regorafenib	Cabozantinib	58:28	68:68	53/5:24/4	Second-line therapy
2	Bang 2021	Retrospective study	Regorafenib	physician’s choice	99:99	/	/	Second-line therapy
3	Bruix 2017	RCT	Regorafenib	Placebo	379:194	64:62	333/46:171/23	Second-line therapy
4	Finn 2018	RCT	Regorafenib	Placebo	379:194	64:62	333/46:171/23	Second-line therapy
5	Casadei-gardini 2021	Retrospective Study	Regorafenib	Cabozantinb	278:331	/	222/46:264/67	Second-line therapy
6	Choi 2020	Retrospective study	Regorafenib	Nivolumab	223:150	58.5 ± 9.4:56.9 ± 10	202/21:125/25	Second-line therapy
7	Iavarone 2021	Retrospective Study	Regorafenib	Best supportive care (BSC)	36:45	60:61	27/9:38/7	Second-line therapy
8	Kuo 2021	Retrospective study	Regorafenib	Nivolumab	58:32	63.4 ± 10.7:62.2 ± 10.1	44/14:23/9	Second-line therapy
9	Lee 2020	Retrospective cohort study	Regorafenib	Nivolumab	102:48:00	62:61	83/54:39/9	Second-line therapy
10	Lee 2024	Retrospective cohort study	Regorafenib	Nivolumab	137:52:00	63:59:00	120/17:45/7	Second-line therapy

### 3.3. Overall survival

**Figure 3.**
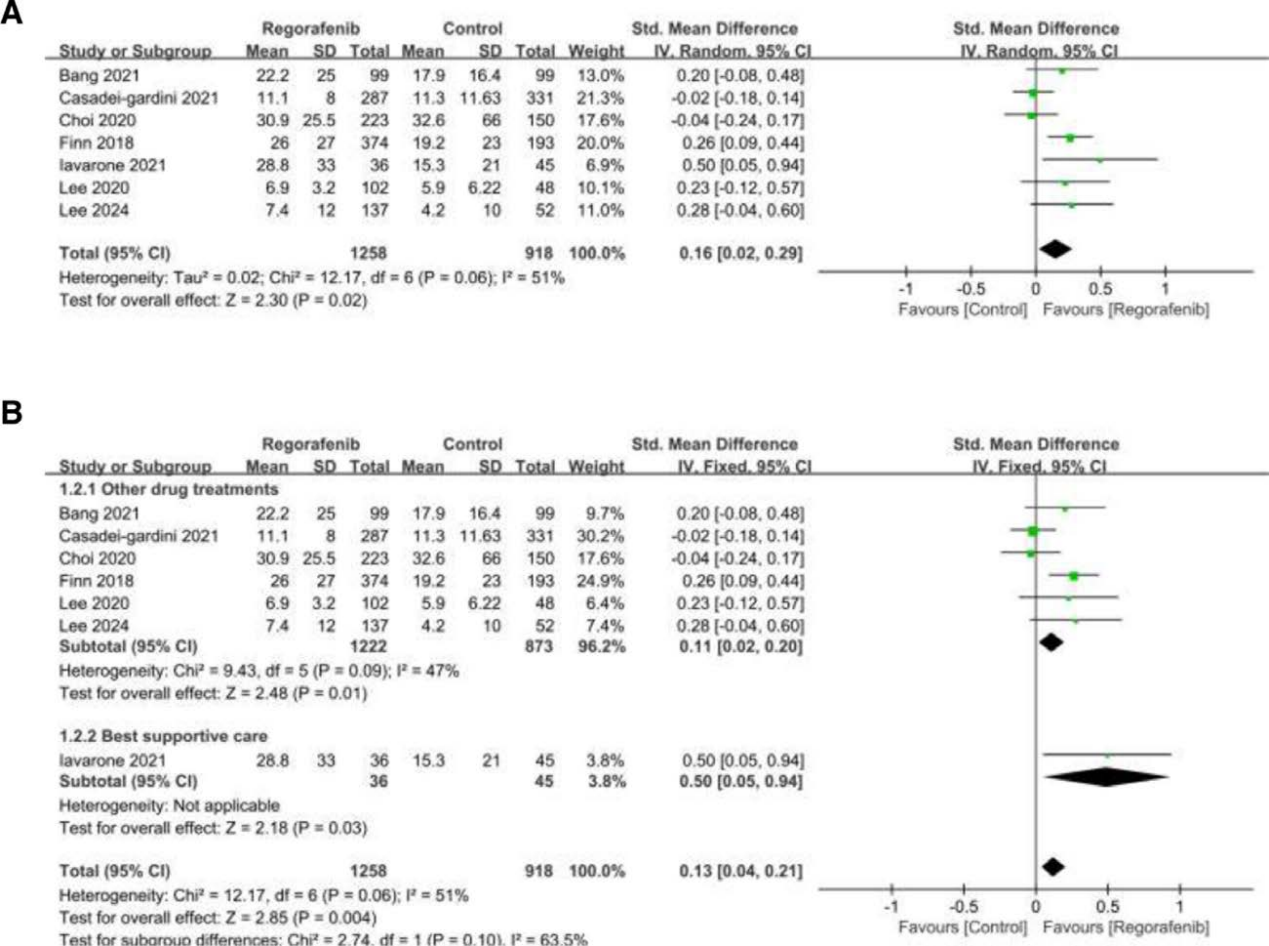
(A) Forest plot of studies for overall survival (OS); (B) Subgroup analysis for OS.

Overall survival was reported in 7 studies. Among them, significant heterogeneity was observed (*I*^2^ = 51%; heterogeneity *P* = .006; Figure 3A). The random-effects meta-analysis showed regorafenib have significantly longer OS than control group (SMD = 0.16, 95% CI: 0.02–0.29, *P* = .02).

To better analyze the OS results, we divided the control group into other drug treatment group (cabozantinb, nivolumab) and best supportive care (BSC) group. Subgroup analysis showed that a statistically significant difference in OS between regorafenib and the other drug treatment group (cabozantinb, nivolumab), with regorafenib having longer OS. The pooled SMD was 0.11 (95% CI: 0.02–0.2, *P* = .01; Figure 3B). The heterogeneity of the included articles was middle (*I*^2^ = 47%, heterogeneity *P* = .09). Only 1 article^[21]^ reported the comparison between regorafenib and BSC therapy, and the results demonstrated that regorafenib is an effective second-line treatment after sorafenib in patients with HCC recurrence (OS, 28.8 months vs 15.3 months, *P* < .01).

### 3.4. Progression-free survival

**Figure 4.**
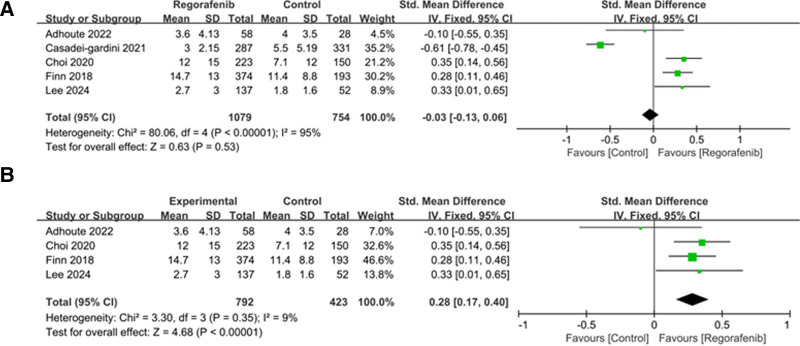
(A) Forest plot of studies for progression-free survival (PFS). (B) Sensitivity analysis of PFS.

PFS was reported in 5 studies. Among them, significant heterogeneity was observed (*I*^2^ = 95%; heterogeneity *P* < .00001; Figure 4A). Random effect model was used and the result showed that no statistical significance between regorafenib and other drug treatment group (cabozantinb, nivolumab) (SMD = −0.03; 95% CI = −0.13 to 0.06; *P* = .53).

Sensitivity analysis demonstrated that Casadei-gardini 2021^[20]^ exit high heterogeneity (Figure S1, Supplemental Digital Content, http://links.lww.com/MD/O303). By omitting Casadei-gardini 2021,^[20]^ the level of heterogeneity is significantly reduced (*I*^2^ = 9%, *P* = .35, Figure 4B), as evidenced by the SMD of 0.28 (95% CI = 0.17–0.4, *P* < .00001). This could potentially explain the high level of heterogeneity observed.

### 3.5. Overall response rate

**Figure 5.**
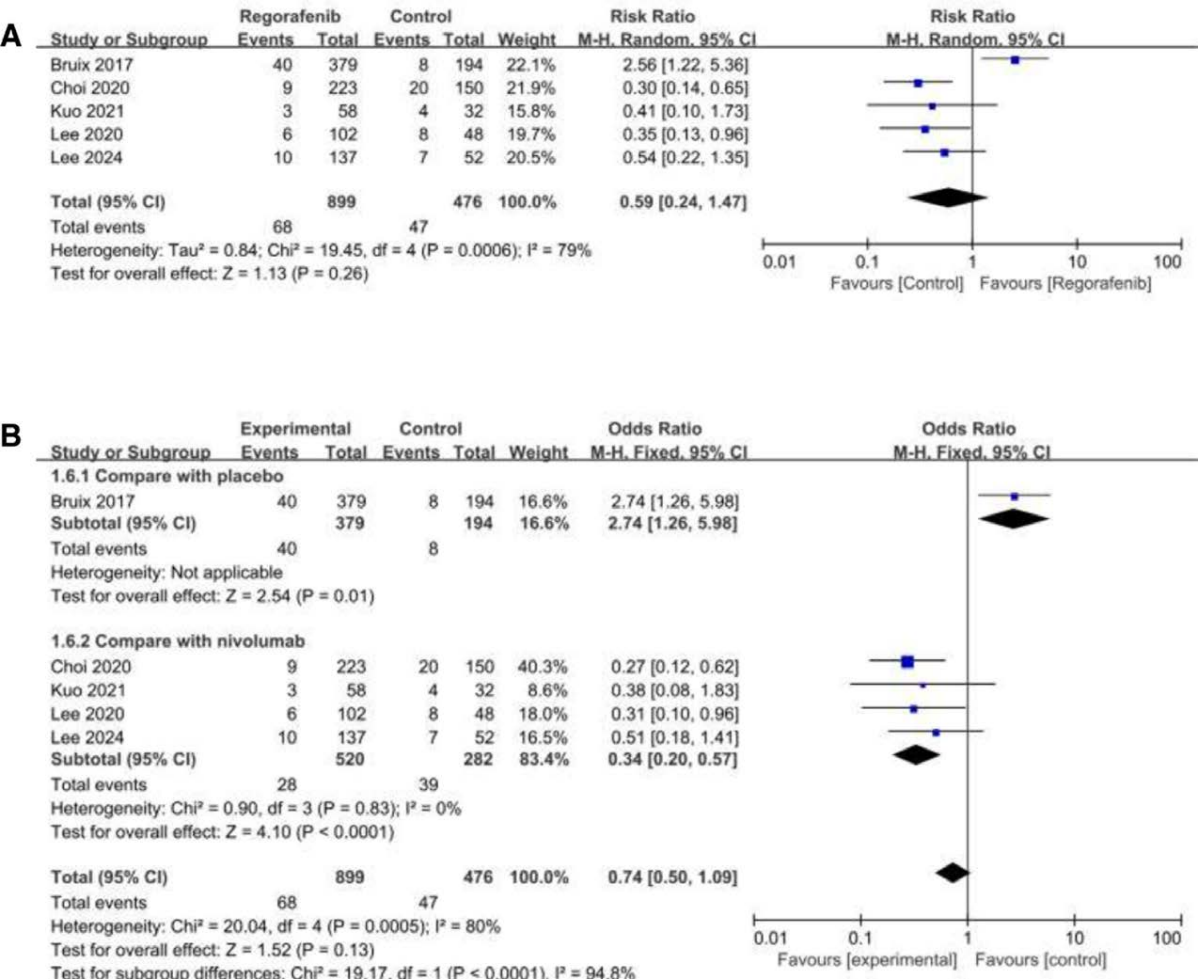
Forest plot of studies for overall response rate (ORR); (B) Subgroup analysis for ORR.

ORR was reported in 5 studies. Among them, significant heterogeneity was observed (*I*^2^ = 80%; heterogeneity *P* = .0005; Figure 5A). Random effect model was used and the result showed that no statistical significance between 2 groups (RR = 0.59; 95% CI = 0.24 to 1.47; *P* = .26).

Subgroup analysis showed that a statistically significant difference in ORR between regorafenib and nivolumab, with nivolumab having higher ORR. The pooled RR was 0.38 (95% CI: 0.24–0.61, *P* < .0001; Figure 5B) with no heterogeneity (*I*^2^ = 0, heterogeneity *P* = .81).

### 3.6. Disease control rate

**Figure 6.**
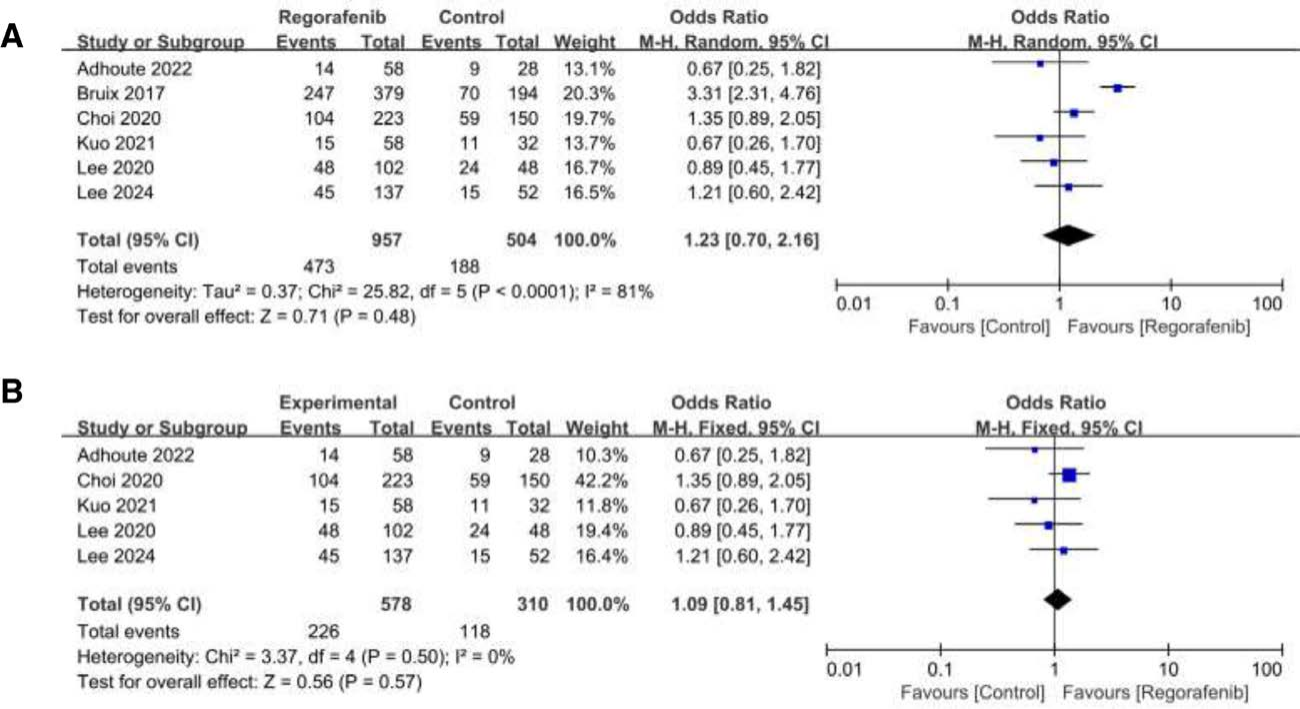
Forest plot of studies for disease control rate (DCR). (B) Sensitivity analysis of DCR.

DCR was reported in 6 studies. Among them, significant heterogeneity was observed (*I*^2^ = 81%; heterogeneity *P* < .0001; Figure 6A). Random effect model was used and the result showed that no statistical significance between 2 groups (RR = 1.23; 95% CI = 0.7 to 2.16; *P* = .48).

Sensitivity analysis demonstrated that Bruix 2017^[12]^ exit high heterogeneity (Figure S2, Supplemental Digital Content, http://links.lww.com/MD/O303). By omitting Bruix 2017, the level of heterogeneity is significantly reduced (*I*^2^ = 0%, *P* = .5; Figure 6B), as evidenced by the SMD of 1.09 (95% CI = 0.81–1.45, *P* = .57). This could potentially explain the high level of heterogeneity observed.“

## 4. Discussion

The treatment of liver cancer has undergone significant changes, moving away from ineffective cytotoxic chemotherapy to the use of anti-angiogenic tyrosine kinase inhibitors. These inhibitors have proven to be beneficial in improving the survival rates of patients with advanced forms of the disease.^[24]^ For several years, sorafenib has remained the only drug approved for advanced or metastatic HCC patients. In second-line therapy, several drugs have recently been approved. For example, in April 2017, regorafenib was approved for clinical practice as the first second-line treatment for HCC.^[12]^ This was soon followed by cabozantinib, ramucirumab, immunotherapeutic drugs nivolumab and pembrolizumab. However, the real efficacy and safety of sorafenib-regorafenib sequential therapy compared with placebo or other treatment (cabozantinib or nivolumab) in advanced HCC regorafenib is still unknown.

Regorafenib is a multiple protein kinase inhibitor that target angiogenesis (the VEGFR 1–3 and the angiopoietin 1 receptor TIE2) more intensively than sorafenib, tumor cells (especially the oncogenic kinases Kinase Insert Domain Receptor (KIT) and Rearranged during Transfection and the intracellular kinases Raf), and fibroblast growth factor receptors, in a more targeted manner than sorafenib.^[25]^ Regorafenib has only 1 additional fluorine atom compared to sorafenib, resulting in a 3-fold increase in its bioavailability. Although regorafenib and sorafenib have overlapping targets, regorafenib targets a wider range of kinases and has stronger inhibitory effects on key angiogenesis receptor targets, such as VEGFR-2, PDGFR-β, fibroblast growth factor receptors-1, and c-Kit. Meanwhile, regorafenib can also inhibit Tie-2, which has a broader antiangiogenesis effect.

The results of our systematic review and meta-analysis found regorafenib could significantly prolong OS than other treatment (cabozantinib or nivolumab or BSC) in the second-line treatment of HCC following sorafenib failure. No significant difference in PFS, ORR or DCR between regorafenib other treatment. Subgroup analysis demonstrated that nivolumab has better ORR results than regorafenib (*P* < .0001).

Our research is like many previous research results. Delos Santos and his colleagues^[26]^ compared 2 indirect networks, demonstrated that cabozantinib, compared with regorafenib, showed similar OS (HR = 1.21), PFS (HR = 1.02) and ORR (RD = −3.0%). And both treatments showed similar toxicities. Recently, Jianxin Chen et al^[27]^ conducted a study that included several comparative trials to investigate the effectiveness of targeted therapy following treatment with sorafenib. Their network meta-analysis revealed that in patients with advanced HCC and elevated AFP levels (400 ng/mL or higher), there were no significant differences in PFS, OS, ORR, or DCR when comparing regorafenib, cabozantinib, or lamuximab.

A 2017 meta-analysis by Kim et al^[28]^ found that second-line targeted therapy, such as regorafenib, significantly improved progression time (*P* < .0001) and showed a trend towards improved OS (*P* = .06). However, it is important to note that this analysis only compared targeted therapy to optimal supportive care, and did not provide guidance for clinicians in choosing between different targeted therapies. Afterwards, Bakouny et al conducted a meta-analysis^[29]^ which reported that among various second-line treatments, regorafenib and cabozantinib showed the best efficacy and safety. However, most of the other treatments did not show better efficacy than placebo. In contrast, our study restricted comparison to second-line treatments that have been proven to have survival benefits, and found similar efficacy and toxicity characteristics between regorafenib and cabozantinib. Therefore, in the absence of a randomized controlled trial directly comparing the 2 regimens, our study can provide valuable insights into the effectiveness and safety of established second-line treatments.

Nowadays, the first-line treatment of HCC is rapidly evolving. In addition to sorafenib, several other therapeutic drugs have also been approved for first-line treatment of liver cancer, such as lenvatinib,^[30]^ atezolizumab + bevacicumab^[31]^ and durvalumab + tremelimumab.^[32]^ There are many treatment options available, and in the future, fewer and fewer patients will choose sorafenib as their first-line treatment. We hope the results of the present study could provide some implication in clinical practice.

The limitations of this study are the limited sample size and retrospective design, which hinder the clear conclusions about the model. However, our results are consistent with previous published articles, and other post-sorafenib treatment studies also included similar population sizes. Due to limited response rates to first-line TKIs and a typical time frame of <6 months to achieve control, only a small number of patients are able to complete second-line therapy.

## 5. Conclusions

In this meta-analysis, compared with other treatment (cabozantinib or nivolumab or placebo), regorafenib seemed to be more effective in patients with HCC after failure of first-line treatment with sorafenib. We hope the results of the present study could provide some implication in clinical practice.

## Author contributions

**Conceptualization:** Yunzhi Shen.

**Data curation:** Yu Bai.

**Software:** Yunzhi Shen, Yu Bai.

**Writing – review & editing:** Yunzhi Shen, Yu Bai.

## Supplementary Material


