# Supplementary material for: Effectiveness of regorafenib in second-line therapy for advanced hepatocellular carcinoma: A systematic review and meta-analysis
Source: Medicine (Baltimore). 2025 Jan 24;104(4):e41356. doi: 10.1097/MD.0000000000041356 (PMC11771663; doi:10.1097/MD.0000000000041356)
Supplement: Supplementary file 1 [file medi-104-e41356-s001.pdf]

Effectiveness of Regorafenib in second-line therapy for advanced hepatocellular carcinoma: a systematic review and meta-analysis

Running title: Effectiveness of Regorafenib for hepatocellular carcinoma

Yunzhi Shen, BS[1234], Yu Bai, MS[1234]

[1]Department of Hepatobiliary Surgery, The Third Central Hospital of Tianjin, Tianjin, China

[2]Tianjin Key Laboratory of Extracorporeal Life Support for Critical Diseases, Tianjin, China

[3]Artificial Cell Engineering Technology Research Center, Tianjin, China

[4]Tianjin Institute of Hepatobiliary Diseases, Tianjin, China

Correspondence address:

Yunzhi Shen, Department of Hepatobiliary Surgery, The Third Central Hospital of Tianjin, No.83, Jintang Road, Hedong District, Tianjin, 300170, China.

Tel: +86-022-84112054; Fax: +86-022-84112054; E-mail: 19902090575@163.com

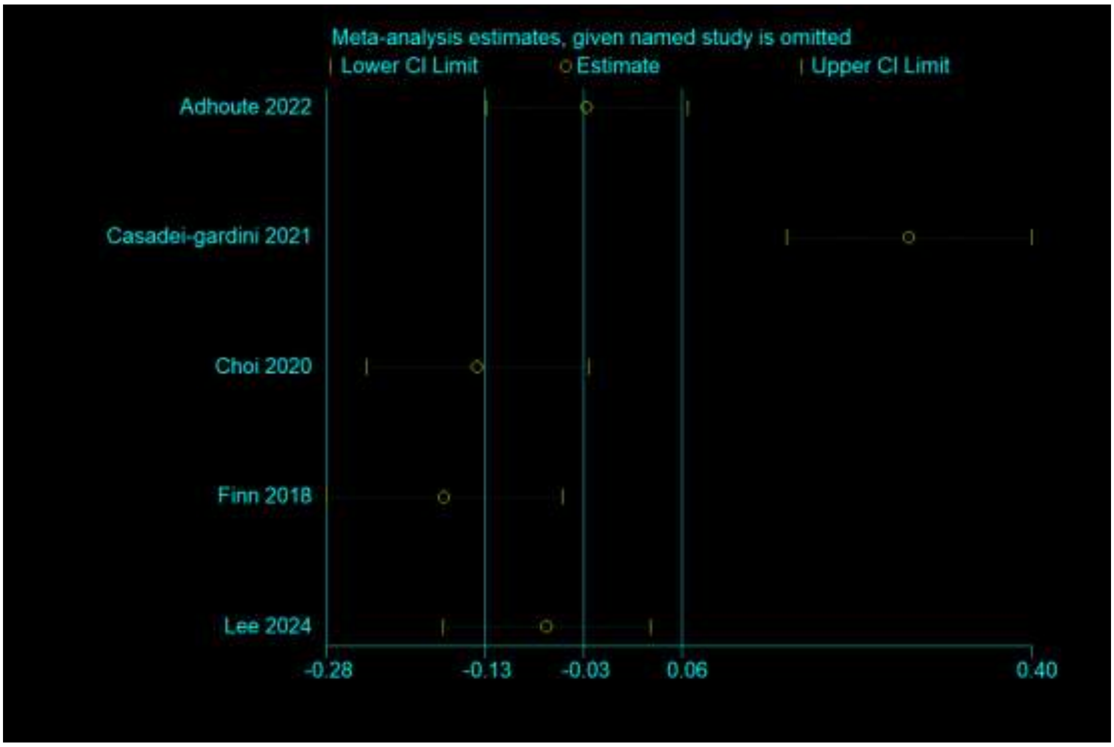

Supplement Figure 1. Sensitivity analysis of PFS.

Effectiveness of Regorafenib in second-line therapy for advanced hepatocellular carcinoma: a systematic review and meta-analysis

Running title: Effectiveness of Regorafenib for hepatocellular carcinoma

Yunzhi Shen, BS[1234], Yu Bai, MS[1234]

[1]Department of Hepatobiliary Surgery, The Third Central Hospital of Tianjin, Tianjin, China

[2]Tianjin Key Laboratory of Extracorporeal Life Support for Critical Diseases, Tianjin, China

[3]Artificial Cell Engineering Technology Research Center, Tianjin, China

[4]Tianjin Institute of Hepatobiliary Diseases, Tianjin, China

Correspondence address:

Yunzhi Shen, Department of Hepatobiliary Surgery, The Third Central Hospital of Tianjin, No.83, Jintang Road, Hedong District, Tianjin, 300170, China.

Tel: +86-022-84112054; Fax: +86-022-84112054; E-mail: 19902090575@163.com

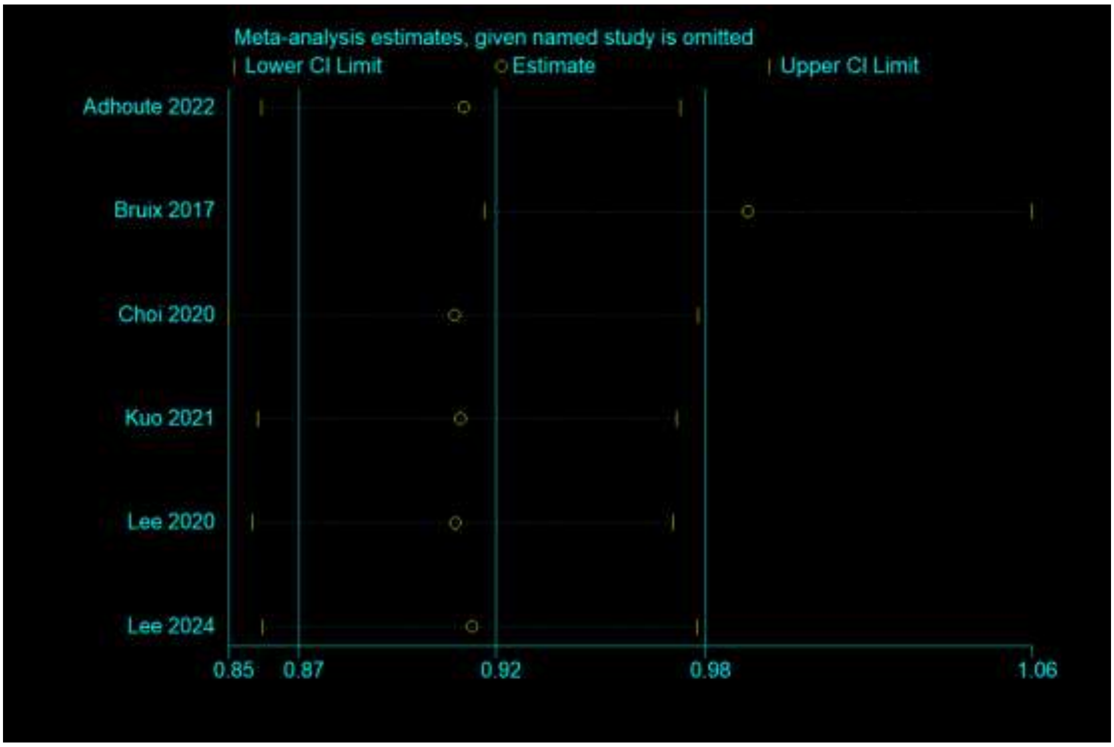

Supplement Figure 2. Sensitivity analysis of DCR.
